# Individuelle computergestützte 3D-Planung zur Platzierung von Epithesenankern in Kombination mit einem implantierbaren transkutanen Knochenleitungshörgerät bei Patienten mit Ohrfehlbildungen

**DOI:** 10.1007/s00106-022-01189-3

**Published:** 2022-08-03

**Authors:** Ingmar Seiwerth, Sebastian Plößl, Michael Herzog, Sebastian Schilde, Florian Radetzki, Steffen Krämer, Torsten Rahne, Stefan K. Plontke

**Affiliations:** 1 Universitätsklinik und Poliklinik für Hals-Nasen-Ohrenheilkunde, Kopf- und Halschirurgie, Martin-Luther-Universität Halle-Wittenberg, Ernst-Grube-Str. 40, 06120 Halle (Saale), Deutschland; 2 Klinik für Hals-Nasen-Ohrenheilkunde, Kopf- und Halschirurgie, Plastische Operationen, Martha-Maria Krankenhaus Halle-Dölau, Halle (Saale), Deutschland; 3 Klinik für HNO-Krankheiten, Kopf- und Halschirurgie, Carl-Thiem-Klinikum Cottbus, Cottbus, Deutschland; 4 Department für Orthopädie, Unfall- und Wiederherstellungschirurgie, Martin-Luther-Universität Halle-Wittenberg, Universitätsklinikum Halle (Saale), Halle (Saale), Deutschland; 5 Klinik für Orthopädie und Unfallchirurgie, Städtisches Klinikum Dessau, Medizinische Hochschule Brandenburg Theodor Fontane, Dessau, Deutschland; 6 MASK-Anaplastologen GmbH, Leipzig, Deutschland

**Keywords:** Schallleitungsschwerhörigkeit, Hörgeräte, Computersimulation, Knochenleitung, Ohrmuschel, Conductive hearing loss, Hearing aids, Computer simulation, Bone conduction, Auricle

## Abstract

**Hintergrund:**

Die simultane Versorgung mit der Bonebridge (MED-EL, Innsbruck, Österreich), einem teilimplantierbaren, aktiven transkutanen Knochenleitungshörgerät, sowie mit Ankern für Ohrepithesen kann eine Herausforderung darstellen, da beide Implantate Magnete enthalten und um ein begrenztes Areal im vorgesehenen Implantationsgebiet konkurrieren.

**Material und Methoden:**

Die Bestimmung der optimalen Implantatposition für den Massenschwingers („floating mass transducer“, FMT) und die Epithesenanker erfolgte mittels einer Software zur 3‑dimensionalen (3D-)Planung („virtuelle Chirurgie“) anhand individueller 3D-Computermodelle der Schädel und der Implantate. Die Interaktion zwischen den magnetischen Epithesenankern und dem FMT wurde mittels statischer Magnetkräfte gemessen. In einer retrospektiven Datenanalyse wurden chirurgische und audiologische Ergebnisse evaluiert.

**Ergebnisse:**

Zwischen den Jahren 2014 und 2021 wurde an 6 Ohren bei 5 Patienten (männlich: *n* = 3, weiblich: *n* = 2, Alter: 17–56 Jahre) die 3D-Planung einer simultanen Implantation der Bonebridge und von Ohrepithesenankern durchgeführt. Die individuelle präoperative Planung war hinsichtlich der optimalen Platzierung von Epithesenankern in Kombination mit der Bonebridge äußerst hilfreich. Audiologisch zeigte sich 3 Monate und > 11 Monate postoperativ ein klarer Nutzen. Es konnten keine Interaktionen zwischen den magnetischen Epithesenankern und dem FMT nachgewiesen werden. Bei 2 Patienten musste aufgrund einer Wundinfektion bzw. aufgrund von Wundheilungsstörungen eine Revisionsoperation erfolgen. Es wurden keine Langezeitkomplikationen (3–5 Jahre postoperativ) beobachtet.

**Diskussion:**

Die präoperative 3D-Planung stellt einen deutlichen Nutzen bei der simultanen audiologischen und ästhetischen Rehabilitation mithilfe der Bonebridge und Ohrepithesenankern dar.

## Abstract (en)

**Background:**

The simultaneous implantation of the Bonebridge (MED-EL, Innsbruck, Austria), a semi-implantable active transcutaneous bone conduction hearing device and anchors for auricular prostheses can be challenging as both implants contain magnets and compete for the narrow space in the designated implantation area.

**Material and methods:**

A preoperative planning tool (virtual surgery) was used with individual 3D computer models of the skull and implants for finding optimal implant positions for both the floating mass transducer (FMT) and the anchors for the auricular prosthesis. The interaction between the magnetic prosthesis anchors and the FMT was measured by means of static magnetic forces. A retrospective data analysis was conducted to evaluate the surgical and audiological outcome.

**Results:**

Between 2014 and 2021 a 3D planning of a simultaneous implantation of the Bonebridge with auricular prosthesis anchors was conducted on 6 ears of 5 patients (3 males, 2 females; age range 17–56 years). The individual preoperative planning was considered very useful for the optimal placement of bone anchors in combination with the Bonebridge. Audiological data showed a clear benefit for hearing 3 months and > 11 months after implantation. No adverse interactions between the magnetic prosthesis anchors and the FMT were observed. In two patients, revision surgery was carried out due to skin inflammation or wound healing problems. No long-term complications were observed 3–5 years after surgery.

**Conclusion:**

Preoperative 3D planning represents a clear benefit for the simultaneous audiological and esthetic rehabilitation using the Bonebridge and anchors for auricular prostheses.

## Hintergrund

Fehlbildungen des äußeren Ohrs und des Mittelohrs gehen oft mit einer Schallleitungsschwerhörigkeit der betroffenen Seite einher [1], sodass sich hier sowohl eine audiologische als auch eine kosmetische Rehabilitation anbietet. Abhängig vom Ausprägungsgrad der Fehlbildung stellt ein kosmetischer Ohrmuschelaufbau mit Versuch einer audiologischer Rehabilitation mittels Tympanoplastik in der Regel eine mehrzeitige, komplexe und anspruchsvolle Prozedur dar [26, 27]. Insbesondere nach erfolglosen Rekonstruktionsversuchen des Mittelohrs und des Gehörgangs stellen implantierbare elektronische Hörgeräte inzwischen eine gängige Methode der Hörrehabilitation dar.

Die simultane Implantation perkutaner knochenverankerter Hörgeräte wie das Baha-System (Cochlear Bone Anchored Solutions, Göteborg, Schweden; [14]) in Kombination mit Ohrepithesen wurde bereits beschrieben [10, 11]. Allerdings kann das Baha-System durch die perkutane Situation mit einem erhöhten Risiko für Wundheilungsstörungen assoziiert sein [15] und weist eine deutlich höhere Komplikationsrate als andere Knochenleitungshörimplantate auf [23].

Im Jahr 2012 wurde Bonebridge („bone conduction implant“ [BCI] 601), ein teilimplantierbares, aktives, transkutanes Knochenleitungshörgerät von der Firma MED-EL (Innsbruck, Österreich; [31]) vorgestellt. Die Haut wird hier durch das Implantat nicht penetriert. Die Informationen werden vom Audioprozessor mittels Induktion zum Implantat transferiert [18].

Die Informationen werden vom Audioprozessor mittels Induktion zum Implantat transferiert

Aufgrund der anatomischen Gegebenheiten kann die chirurgische Platzierung des Massenschwingers („floating mass transducer“, FMT: Tiefe 8,7 mm, Durchmesser 15,8 mm), der aktiven, die akustische Energie übertragenden Einheit der Bonebridge, vor dem Hintergrund der Knochenstärke und der Lage der Dura oder des Sinus sigmoideus eine Herausforderung darstellen. Dies kann insbesondere bei Kindern mit kleinen Mastoiden, bei Malformationen und nach vorhergehender Ohrchirurgie der Fall sein [19, 21]. Falls erforderlich kann die Implantationstiefe durch die Verwendung von BCI Lifts reduziert werden. Seit September 2019 ist ein Nachfolgemodell (BCI 602, MED-EL, Innsbruck, Österreich) mit veränderten FMT-Maßen (Tiefe 4,5 mm, Durchmesser 18,2 mm) in Europa verfügbar [16, 34].

Bei Patienten mit angeborenen Fehlbildungen des Mittelohrs und des äußeren Ohrs – oft mit einer Vorgeschichte von in der Regel in der Kindheit erfolgten, frustranen Versuchen einer Ohrmuschelrekonstruktion – besteht die Möglichkeit der kosmetischen Rehabilitation mittels einer knochenverankerten Ohrepithese [9]. Diese Epithesen stellen eine sichere Methode für die Langzeitverankerung mit Erfolgsraten hinsichtlich der Implantatüberlebensrate zwischen 95 % und 99 % dar [9].

Der FMT und die Ohrepithesenanker konkurrieren um ein begrenztes chirurgisches Implantationsareal

Im Fall einer gleichzeitigen audiologischen Rehabilitation mit Implantation eines transkutanen, teilimplantierbaren knochenverankerten Hörgeräts kann sich die Situation komplexer darstellen, da der FMT und die Ohrepithesenanker (wie auch deren Magnete) um ein begrenztes chirurgisches Implantationsareal konkurrieren. Umso mehr erscheint es wichtig, die Platzierung der Bonebridge und der Epithesenanker optimal zu planen. In dieser Studie wurde eine in der Klinik der Autoren etablierte 3‑dimensionale (3D-)Planungsmethode [17] im Sinne der Planung einer simultanen Platzierung von Ohrepithesenankern in Kombination mit dem Bonebridge-System weiterentwickelt.

Ziel ist es hier, die chirurgischen und funktionellen Ergebnisse der simultanen Platzierung von Ankern für individuelle Ohrepithesen in Kombination mit dem Bonebridge-System zu evaluieren.

## Methoden

Diese retrospektive Studie wurde mit Genehmigung (No. 2019-123) der Ethikkommission der Medizinischen Fakultät der Martin-Luther-Universität Halle-Wittenberg durchgeführt. In einer retrospektiven Datenanalyse wurden alle Patienten eingeschlossen, bei denen im Zeitraum von Januar 2014 bis November 2021 die simultane Planung einer Bonebridge- und Ohrepithesenversorgung erfolgte.

### Präoperative Planungssoftware

Basierend auf dem Digital-Imaging-and-Communications-in-Medicine(DICOM)-Datensatz von Dünnschicht-Computertomographie(CT)-Aufnahmen wurde ein 3D-Modell des Schläfenbeins mittels der Visualisierungssoftware Amira (Versionen 5.2–6.3, FEI Visualization Sciences Group, Burlington, MA, USA) rekonstruiert. In einem zweiten Schritt wurden 3D-Modelle des FMT und der Epithesenanker mit dem 3D-Modell des jeweiligen Schläfenbeins fusioniert. Im Sinne einer „virtuellen Chirurgie“ konnten alle Komponenten unabhängig voneinander frei im Raum bewegt werden, bis die optimalen Implantationsorte für den FMT und die Epithesenanker bestimmt werden konnten.

Alle Komponenten können unabhängig voneinander frei im Raum bewegt werden

**Figure 1:**
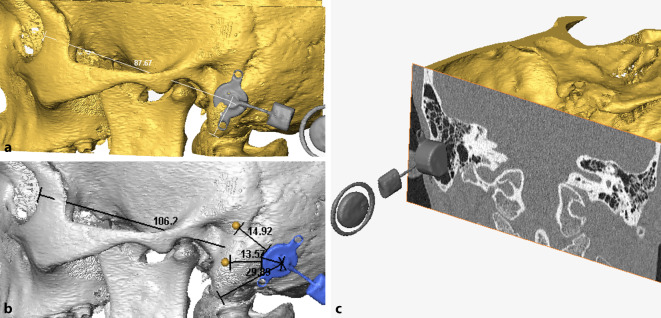

**Figure 2:**
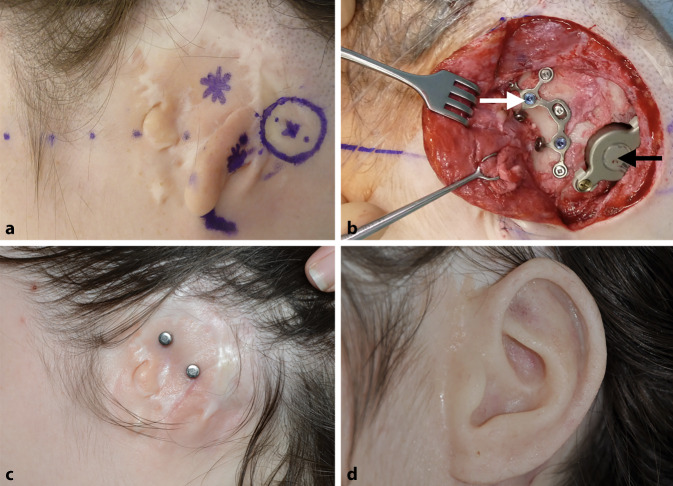

Zudem bestand in allen 3D-Ansichten die Möglichkeit, in das Modell die jeweiligen axialen, sagittalen oder koronaren CT-Schichten einzublenden. Auf diese Weise konnten die designierten Positionen des FMT und der Epithesenanker aus allen perspektiven hinsichtlich einer korrekten Lage und möglicher intrakranieller Impressionen (Dura oder Sinus sigmoideus) überprüft werden. Zur intraoperativen Identifikation der optimalen Implantatposition wurden die Abstände vom Mittelpunkt des FMT und der Anker zu anatomischen Landmarken (in der Regel der Jochbogenansatz, die Mastoidspitze und der laterale Orbitarand) im 3D-Modell gemessen (Abb. 1b). Die intraoperativ korrekte Position des Implantats und der Epithesenanker wurde mittels der Schnittpunkte der jeweiligen Distanzen ermittelt (Abb. 2a).

### Operation

In einem ersten Schritt wurden, sofern erforderlich, Rudimente der Ohrmuschel oder Narbengewebe von früheren Operationen (die üblicherweise in der Kindheit durchgeführt wurden) reseziert. Die geplanten Positionen des FMT und der Epithesenanker wurden entsprechend der präoperativen 3D-Planung markiert (Abb. 1b und 2a). Nach dem Hautschnitt erfolgte das Fräsen des Implantatbetts ggf. mit Skelettierung der Dura und des Sinus sigmoideus als chirurgische Landmarken. Auch wenn eine leichte Impression der Dura oder des Sinus sigmoideus akzeptabel ist, wurde versucht, dies mittels präoperativer 3D-Planung zu vermeiden. Für den Fall des Überschreitens einer Impressionstiefe von 1–2 mm wurde diese durch Unterlegen von BCI Lifts etwas reduziert. Im Anschluss wurden die Epithesenanker (Epiplating plate system, Medicon, Tuttlingen, Deutschland) entsprechend der präoperativen 3D-Planung positioniert und festgeschraubt. Nach dem Wundverschluss erfolgte die Platzierung von „Heilkappen“, die im Verlauf nach Abschluss der Wundheilung durch Magnete ersetzt wurden.

### Anaplastologie

Die individuelle Anfertigung von Ohrepithesen erfolgte durch einen Anaplastologen (SK). Als Material wurde Silikon („medical grade“ 2) mit Hartheitsgraden von 10–30 Shore verwendet. Die verwendeten Magnete (steco-system-technik, Hamburg, Deutschland) zeichneten sich durch Abzugskräfte zwischen 1,4–3,0 N aus.

### Magnetinteraktion und Audiologie

Zur Untersuchung einer magnetischen Wechselwirkung wurde die Interaktion zwischen den Magneten der Epithese und dem FMT, der ebenfalls einen beweglichen Magneten zur Übertragung der Vibrationsenergie auf den Schädelknochen enthält, erfasst. Hierfür wurden statische Magnete, wie sie bei der Epithese verwendet werden, an verschiedenen Stellen auf der Haut in einem Umkreis von 4 cm um den FMT sowie an den finalen Positionen der Epithesenanker angelegt. Die Stimulation des Sprachprozessors erfolgte jeweils mittels aktiver Sprachstimulation und die Klangqualität wurde subjektiv vom Patienten bewertet. Im Fall einer Interaktion würde eine reduzierte Stimulation in einer Abnahme des maximalen Ausgangspegels resultieren, was sich wiederum in einer Reduktion der dynamischen Breite oder einer erhöhten Verzerrung widerspiegeln würde.

### Audiologische Messungen

Die Bestimmung von Reintonhörschwellen für die betroffene Seite erfolgte entsprechend der klinischen Routinediagnostik für Luftleitung (LL) und Knochenleitung (KL), während für die Messung postoperativ versorgter Hörschwellen Wobble-Töne verwendet wurden.

Das Sprachverstehen in Ruhe bei 65 dB Schalldruckpegel (SPL; Word Recognition Score, WRS_65_) wurde 1–3 Monate und > 11 Monate postoperativ unter Verwendung des Freiburger Sprachtests erfasst. Das Sprachverstehen im Störschall wurde mit dem Oldenburger Satztest in bestversorgter und unversorgter Situation mit frontaler Präsentation von Sprache und Störschall (S_0_N_0_) gemessen.

## Ergebnisse

### Demographische Daten

Zwischen 2014 und 2021 erfolgte bei 6 Ohren von 5 Patienten (männlich: *n* = 3, weiblich: *n* = 2, Altersdurchschnitt 43,6 Jahre, Spanne von 17–56 Jahren) die präoperative 3D-Planung von Ohrepithesen und simultaner Bonebridge-Implantation im Sinne einer „virtuellen Chirurgie“. Die audiologischen Daten einiger dieser Patienten wurde bereits in einer früheren Studie als Teil einer größeren Patientengruppe vorgestellt [24].

Alle Patienten hatte eine Vorgeschichte von mehrfachen, frustranen Rekonstruktionsversuchen der Ohrmuschel, Gehörgangsrekonstruktionen oder Ohroperationen (Tab. 1). Präoperativ wurden alle Patienten im Team des interprofessionellen Hörimplantatzentrums der Autoren besprochen. Hierbei erfolgte auch die Prüfung der Verwendung alternativer Hörrehabilitationsoptionen, wie z. B. perkutaner Knochenleitungshörgeräte oder aktiver Mittelohrimplantate, die jedoch aus unterschiedlichen, individuellen Gründen, wie anatomischer Limitationen oder dem Patientenwunsch folgend, keine Option darstellten. In einem Fall (Patient 3) waren die Epithesenanker bereits in situ, während der FMT der Bonebridge noch an geeigneter Stelle positioniert werden musste. In einem anderen Fall (Patientin 5) erfolgte 5 Jahre zuvor anderenorts eine simultane Epithesen- und Bonebridge-Implantation. Bei Wundinfektion der Epithesenanker und Dislokation des FMT aus dem Knochenbett erfolgte eine präoperative 3D-Planung zum Implantwechsel auf eine BCI 602.

**Table 1:** 

ID	Alter bei OP	M/W	Seite	Diagnose/Indikation	Otologische Vorgeschichte (operiertes Ohr)	FMT-Position	Sinus‑/Dura-Exposition bei Bonebridge-Implantation	Komplikationen
1	17	W	L	Fehlbildung (Mikrotie, Gehörgangsatresie, Hammer- und Ambossdysplasie)	Multiple, frustrane Rekonstruktionen des äußeren Ohrs	RS	Partielle Exposition des Sinus sigmoideus und der Dura, BCI Lifts (2 mm) zur Vermeidung der Impression	Keine
2	51	M	R	Fehlbildung des äußeren Ohrs und des Mittelohrs chronische OE, partielle Paukenfibrose, postinflammatorische Stapesfixation chronische Mastoiditis	Gehörgangs-OP, Anlage einer offenen Mastoidhöhle	SDW	Exposition des Sinus sigmoideus und minimale Impression, BCI Lifts (1 mm)	Partielle Hautnekrose, Revisions-OP 22 Tage post-OP
3	45	M	R	Ohrfehlbildung; Prothesenanker bereits in situ	Gehörgangsanlage; 2 TPL-Revisionen; Implantation und Reimplantation von Epithesenankern	SDW	Minimale Sinusexposition, Abdeckung mit TachoSil^a^	Keine
4	49	M	R	Beidseitige Ohrfehlbildung und Gehörgangsatresie, chronisch-sezernierende OE	Gehörgangsanlage, Radikalhöhlenanlage in der Kindheit	SDW	Minimale Sinusimpression, BCI Lifts (2 mm)	Granulationsgewebe um Prothesenanker: Revisions-OP 2 Monate post-OP
4	49	M	L	Beidseitige Ohrfehlbildung und Gehörgangsatresie, chronisch-sezernierende OE	Gehörgangs-OP, Anlage einer offenen Mastoidhöhle in der Kindheit	SDW	Dura exponiert, minimale Sinusimpression, Abdeckung mit TachoSil^a^	Keine
5	56	W	R	FMT-Protrusion und Dislokation, Hautinfektion Epithesenanker, sezernierende COM, Fehlbildung	Gehörgangs-OP, Epithesenanker- und Bonebridge-Implantation vor 5 Jahren anderenorts	Nur Planung: SDW	*n*.z.	*n*.z.

^a^Takeda, Berlin, Deutschland

*BCI *„bone conduction implant“, *COM* chronische Otitis media, *FMT* „floating mass transducer“ (Massenschwinger), *ID* Identifikationsnummer, *L* links, *M* männlich, *n.z.* nicht zutreffend, *OE* Otitis externa, *OP* Operation, *post-OP* postoperativ, *R* rechts, *RS* retrosigmoidal; *SDW* Sinus-Dura-Winkel; *TPL* Tympanoplastik, *W* weiblich

### Operation

Die Implantation der Bonebridge konnte in allen Fällen ohne Komplikationen durchgeführt werden (Abb. 2, 3 und 4). In einem Fall erfolgte, wie präoperativ geplant, eine retrosigmoidale Implantation des FMT (Patientin 1, Abb. 1), während der FMT bei den anderen 4 Operationen im Sinus-Dura-Winkel (Mastoid) implantiert wurde. In 4 Fällen (3 Patienten) wurden Epithesenanker implantiert. Da die definitive Entscheidung, welche Art der Ankerplatte („Stern“ oder kombinierte Platte) zum Einsatz kommen sollte, erst intraoperativ unter Berücksichtigung der Anatomie gefällt wurde, konnte im Einzelfall die finale Ankerposition von der in der präoperativen 3D-Planung ermittelten Position abweichen. Bei einem Patienten (Patient 3, Abb. 4) waren Epithesenanker bereits vorhanden.

**Figure 3:**
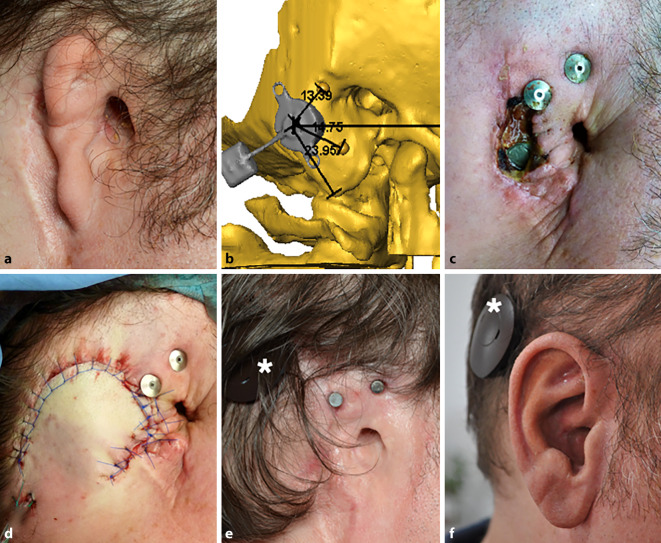

**Figure 4:**
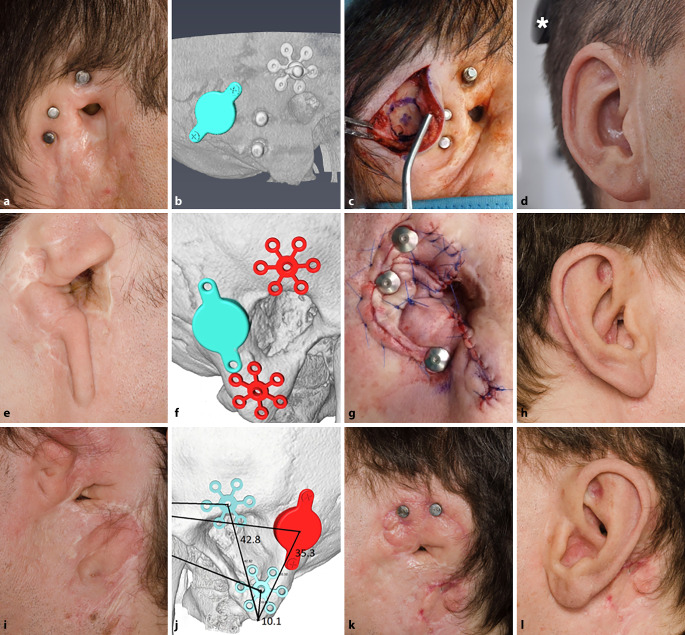

Bei allen BCI-601-Implantationen kam es zu einer Exposition des Sinus sigmoideus oder der Dura

Bei allen 5 Bonebridge-Implantationen (BCI 601) kam es zu einer Exposition des Sinus sigmoideus oder der Dura. In 3 von 5 Fällen war eine minimale Impression des Sinus oder der Dura erforderlich, wie es bereits anhand der präoperativen Planung zu erwarten war, und in 3 Fällen mussten zusätzlich BCI Lifts (1 mm oder 2 mm) verwendet werden, um eine tiefere Impression des Sinus oder der Dura zu vermeiden.

**Figure 5:**
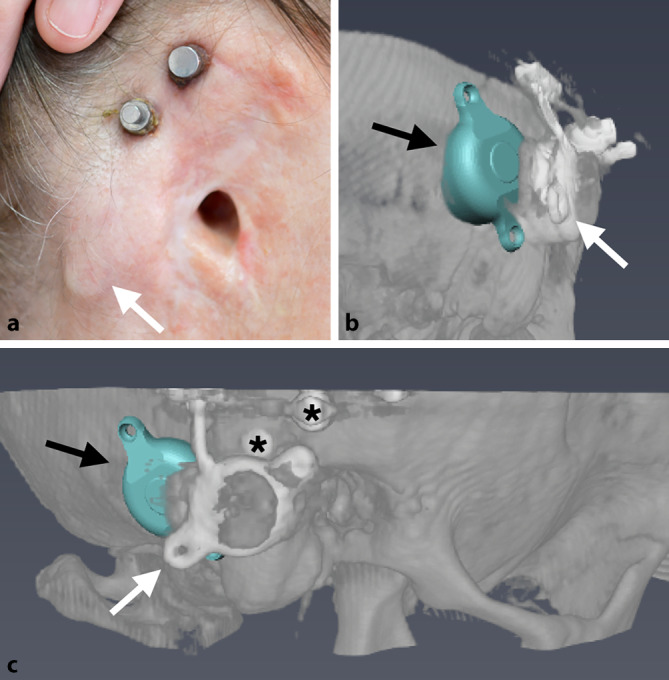

Wie bei Patient 4 auf der rechten Seite zu sehen ist (Abb. 4e–h), war – anders als initial geplant – in der superioren Ankerposition aufgrund der intraoperativen Situation die Implantation von 2 Ankern mittels einer kombinierten Platte erforderlich. Im Fall von Patientin 5 zeigte sich die optimale Implantatposition (BCI 602) unter Berücksichtigung eines Sicherheitsabstands zu den Epithesenankern etwas dorsokaudal der aktuellen Lage des dislozierten FMT (BCI 601). Der insbesondere im kaudalen Anteil aus dem Bett dislozierte FMT war kranial in unmittelbarer Nähe zu den Epithesenankern gelegen (Abb. 5). Direkt präoperativ wurde seitens der Patientin eine erneute Bonebridge-Implantation nicht mehr gewünscht, sodass nur die FMT-Explantation, Duraverstärkung, Wundrevision im Epithesenankerbereich und ein Verschluss des äußeren Gehörgangs mit lokaler Lappenplastik erfolgte. Dementsprechend wurde Patientin 5 von der audiologischen Auswertung ausgenommen. Intraoperativ zeigte sich der FMT kapselartig von Bindegewebe umwachsen und aus seinem Lager herausgekippt mit über dem Knochen schwebender kaudaler Schraubenfixierung wie bereits in der präoperativen Planung ersichtlich (Abb. 5). Sinus und Dura waren zum Teil exponiert und mit dem Bindegewebe verwachsen.

In einem Fall (Patient 2), war aufgrund einer partiellen Hautnekrose (Abb. 3c) im Resektionsareal der Ohrmuschelrudimente eine Revisionschirurgie mittels eines Rotationslappens (Abb. 3d) 22 Tage nach der Operation erforderlich. Bei einem anderen Patienten (Patient 4, rechte Seite) musste 2 Monate postoperativ Granulationsgewebe um den Epithesenanker abgetragen werden. Während des gleichen Eingriffs wurden auch Ohrmuschelrudimente auf der linken Seite entfernt, da auch hier im Rahmen eines späteren Eingriffs eine simultane Bonebridge- und Epithesenankerimplantation geplant war. Bei diesem Patienten wurden aus anaplastologischen Gründen auf beiden Seiten nur die superioren Anker zur Prothesenadhäsion verwendet, wie in Abb. 4k dargestellt ist. Alle Komplikationen konnten erfolgreich behoben werden und es wurden keine weiteren Komplikationen im Nachbeobachtungszeitraum von 3–5 Jahren beobachtet.

### Magnetinteraktion

Es konnten keine Interaktionen zwischen den magnetischen Prothesenankern und dem FMT festgestellt werden. Auch die Veränderung der Abstände und somit der Stärke der potenziell störenden statischen Magnetfelder vom Implantat führte zu keiner Veränderung der Klangqualität. Es kam zu keiner durch die Magnetfelder induzierten Änderung des Hörvermögens, weder mit noch ohne Ohrepithese.

### Audiologische Ergebnisse

**Figure 6:**
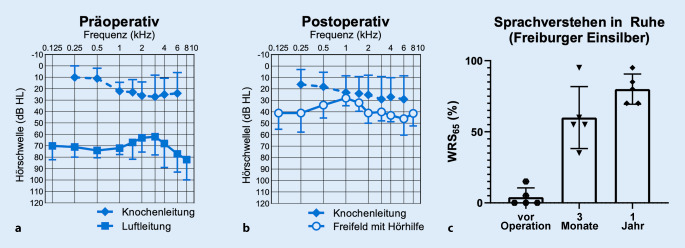

Präoperativ lag die mittlere Reintonhörschwelle (4PTA_0,5–4_ _kHz_) bei 70 dB (Standardabweichung [SD] 9) für Luftleitung und bei 21 dB (SD 7) für Knochenleitung. Die Luftleitung mit Bonebridge verbesserte sich 3 Monate postoperativ auf 39 dB (SD 5) und > 11 Monate postoperativ auf 37 dB HL (SD 6; Abb. 6a, b).

Die mittlere Worterkennungsrate für einsilbige Worte in Ruhe verbesserte sich von präoperativ unversorgt 4 % (SD 6) oder bestversorgt 70 % (SD 16) auf Bonebridge-versorgt

- 60 % (SD 20) 3 Monate postoperativ,
- 80 % (SD 10) > 11 Monate postoperativ (Abb. 6c) und
- 63 % (SD 6) 3–5 Jahre postoperativ.

Die Sprachverständlichkeitsschwelle im Störschall verbesserte sich von −1,4 dB SNR (SD 3) in der unversorgten auf −1,8 dB SNR (SD 3) in der Situation mit Bonebridge.

## Diskussion

Soweit es den Autoren bekannt ist, erfolgt hier erstmals die Vorstellung einer 3D-Planungsmethode („virtuelle Chirurgie“) speziell zur simultanen Implantation der Bonebridge mit Ohrepithesenankern. Im Rahmen einer anderen Fallserie wurde die chirurgische Versorgung mit einer knochenverankerten Ohrepithese und der Bonebridge bei 3 Patienten (4 Ohren) als einzeitiger Eingriff vorgestellt [35]. Dort wurde die Implantatposition 2‑dimensional, basierend auf hochauflösenden CT-Aufnahmen, ermittelt. Eine andere Studie beschreibt eine simultane Bonebridge-Implantation im Rahmen einer einzeitigen Ohrmuschelrekonstruktion mit einem Medpor-Gerüst (Stryker, Kalamazoo, MI, USA) oder als 2‑zeitiger Eingriff mit autologem Material und präoperativer 3D-Planung [4]. Wang et al. beschreiben ebenfalls eine Technik mit autologer chirurgischer Ohrrekonstruktion und Bonebridge-Implantation im Sinne eines 2‑zeitigen Eingriffs [32] und Fan et al. (2017) beschreiben dies als 3‑Schritte-Methode [7]. Während bei Fan et al. eine präoperative 3D-Planung durchgeführt wurde [7], erfolgte bei Wang et al. die Planung 2‑dimensional [32].

Eine präoperative 3D-Planung kann die Sicherheit bei der Bonebridge-Chirurgie erhöhen

Prinzipiell kann eine präoperative 3D-Planung die Sicherheit bei der Bonebridge-Chirurgie erhöhen und es steht eine Vielzahl an mehr oder weniger komplexen Methoden für eine präoperative computergestützte Planung zur Verfügung, um eine optimale – oder zumindest akzeptable – Position für den FMT der Bonebridge zu bestimmen. Eine systematische Übersicht über präoperative Planungsmethoden der Bonebridge-Implantation wurde bereits anderweitig vorgestellt [25]. Die verwendete Planungsmethode sollte, insbesondere im Fall einer zusätzlichen Implantation von Ohrepithesenankern, einen akkuraten Transfer der designierten Implantatpositionen in den Operationssitus gewährleisten.

Da die Epithesenanker und der FMT um die zur Verfügung stehende Implantationsfläche konkurrieren, sind die Möglichkeiten für eine sichere FMT-Platzierung eingeschränkt. Bei Patient 4 stellte sich die Situation nach Gehörgangsoperation und Anlage einer offenen Mastoidhöhle in der Kindheit zusätzlich erschwert dar (Abb. 4f). Hier war es wichtig, eine Protrusion des FMT in die offene Höhle zur vermeiden, um das Risiko von Infektionen im Implantatbereich zu verhindern [3]. Inwieweit bei der Patientin Nr. 5 ein Zusammenhang zwischen der geringen FMT-Anker-Distanz, der Wundinfektion um die Epithesenanker und der FMT-Dislokation existiert, kann nicht sicher beurteilt werden. Die präoperative 3D-Planung ergab in diesem Fall die Möglichkeit einer sicheren FMT-Platzierung, auch wenn dies später von der Patientin nicht mehr gewünscht wurde.

Bei perkutanen Hörgeräten besteht das zusätzliche Risiko von Wundinfektionen

Die Problematik der eingeschränkten Implantationsfläche für den FMT und die Epithesenanker kann durch Verwendung alternativer Möglichkeiten der apparativen Hörrehabilitation, wie z. B. mittels eines perkutanen knochenverankerten Hörsystems oder eines aktiven Mittelohrimplantats, umgangen werden. Allerdings besteht bei perkutanen Hörgeräten das zusätzliche Risiko von Wundinfektionen [15] und die Möglichkeit der Implantation aktiver Mittelohrimplantate kann aus anatomischen oder klinischen Gründen limitiert sein. Zudem gehen diese – insbesondere bei Fehlbildungen [12] – mit einem erhöhten operativen Risiko einher wie z. B. einer Verschlechterung der Knochenleitungshörschwelle [28]. Aktuelle Studien beschreiben hier eine Explantationsrate von 10,2 % [2] und, je nach Ankopplungsort des FMT, Revisionsraten zwischen 10,2 % und 29 % [6, 22, 30].

Aus audiologischer Sicht scheint der Abstand zwischen der Position der Knochenstimulation und der Cochlea die Hörschwelle zu beeinflussen [20]. Bezüglich der Epithesenanker beschreibt Federspil (2010) einen Abstand von 2 cm von der Mitte des äußeren Gehörgangs als ideale Position für Ankerelemente [8]. Diese Aspekte können im Rahmen der präoperativen 3D-Planung gut berücksichtigt werden.

Bei allen 5 Ohren mit Bonebridge und Epithesenankern traten keine Langzeitkomplikationen auf

Bei 2 Patienten war aufgrund einer Hautinfektion oder aufgrund von Wundheilungsstörungen mit partieller Hautnekrose (Abb. 3c) eine Revisionsoperation (Abb. 3d) indiziert. Diese Komplikationen können, müssen aber nicht zwingend mit der simultanen Implantation des knochenverankerten Hörsystems und der Epithesenanker assoziiert sein. Vor dem Hintergrund der Vorgeschichte von – oft multiplen – Voroperationen und konsekutiver, zum Teil ausgeprägter Vernarbung im Operationsgebiet bestand ein höheres Risiko von Wundheilungsstörungen. Bei allen 5 Ohren mit Bonebridge und Epithesenankern (Patientin 5 ist hier nicht eingeschlossen, da eine Bonebridge-Versorgung zwar geplant, jedoch nicht durchgeführt wurde) wurden im Nachbeobachtungszeitraum von 3–5 Jahren keine Langzeitkomplikationen beobachtet.

Bezüglich einer möglichen elektromagnetischen Interaktion zwischen der Bonebridge und den Magnetankern konnten keine nachteiligen Effekte beobachtet werden. Bei allen Patienten zeigte sich postoperativ ein großer funktioneller (audiologischer) Benefit, was sich mit den Ergebnissen andere Studien deckt [5, 13, 29, 31, 33].

Im September 2019 wurde in Europa das Nachfolgemodell BCI 602 mit veränderten Dimensionen eingeführt. Die Implantationstiefe wurde auf 4,5 mm reduziert und es konnte ein besseres Passvermögen in Mastoiden von Kindern und jungen Erwachsenen nachgewiesen werden [16, 34]. Andererseits hat sich der Durchmesser auf 18,2 mm vergrößert, wodurch möglicherweise die Konkurrenzsituation mit den Epithesenankerpositionen auf der zur Verfügung stehenden Oberfläche verschärft wird. Daher wird auch mit dem neuen Modell BCI 602 eine sorgfältige präoperative 3D-Planung bei simultaner Implantation mit Epithesenankern empfohlen.

## Fazit für die Praxis

- Die Simultane Implantation des Bonebridge-Hörsystems mit Ankern für individuelle Ohrepithesen stellt eine adäquate Möglichkeit der gleichzeitigen kosmetischen und audiologischen Rehabilitation dar.
- Eine präoperative 3‑dimensionale Planung kann die optimale Identifikation der jeweiligen Implantationsorte unterstützen.
- Auch wenn die lokalen Gewebsverhältnisse durch Vernarbungen aufgrund von Voroperationen häufig eine Herausforderung darstellen, können die ästhetischen und audiologischen Ergebnisse der kombinierten Rehabilitationsmaßnahme als gut eingeschätzt werden.
- Im Nachbeobachtungszeitraum von 3–5 Jahren wurden keine Langzeitkomplikationen beobachtet.

## References

[1] Bartel-Friedrich S (2015). Congenital auricular malformations: description of anomalies and syndromes. Facial Plast Surg.

[2] Brkic FF, Riss D, Auinger A (2019). Long-term outcome of hearing rehabilitation with an active middle ear implant. Laryngoscope.

[3] Brkic FF, Riss D, Scheuba K (2019). Medical, technical and audiological outcomes of hearing rehabilitation with the Bonebridge transcutaneous bone-conduction implant: a single-center experience. J Clin Med.

[4] Chan KC, Wallace CG, Wai-Yee Ho V (2019). Simultaneous auricular reconstruction and transcutaneous bone conduction device implantation in patients with microtia. J Formos Med Assoc.

[5] Eberhard KE, Olsen SO, Miyazaki H (2016). Objective and subjective outcome of a new transcutaneous bone conduction hearing device: half-year follow-up of the first 12 nordic implantations. Otol Neurotol.

[6] Ernst A, Todt I, Wagner J (2016). Safety and effectiveness of the vibrant soundbridge in treating conductive and mixed hearing loss: a systematic review. Laryngoscope.

[7] Fan X, Wang Y, Wang P (2017). Aesthetic and hearing rehabilitation in patients with bilateral microtia-atresia. Int J Pediatr Otorhinolaryngol.

[8] Federspil PA (2010). Auricular prostheses. Adv Otorhinolaryngol.

[9] Federspil PA (2018). Auricular prostheses in microtia. Facial Plast Surg Clin North Am.

[10] Federspil PA (2015). The role of auricular prostheses (epitheses) in ear reconstruction. Facial Plast Surg.

[11] Federspil PA, Koch A, Schneider MH (2014). Percutaneous titanium implants for bone conduction hearing aids: experience with 283 cases. HNO.

[12] Frenzel H, Sprinzl G, Widmann G (2013). Grading system for the selection of patients with congenital aural atresia for active middle ear implants. Neuroradiology.

[13] Gerdes T, Salcher RB, Schwab B (2016). Comparison of audiological results between a transcutaneous and a percutaneous bone conduction instrument in conductive hearing loss. Otol Neurotol.

[14] Hakansson B, Tjellstrom A, Rosenhall U (1985). The bone-anchored hearing aid. Principal design and a psychoacoustical evaluation. Acta Otolaryngol.

[15] Hobson JC, Roper AJ, Andrew R (2010). Complications of bone-anchored hearing aid implantation. J Laryngol Otol.

[16] Plontke SK, Gotze G, Wenzel C (2020). Implantation of a new active bone conduction hearing device with optimized geometry. HNO.

[17] Plontke SK, Radetzki F, Seiwerth I (2014). Individual computer-assisted 3D planning for surgical placement of a new bone conduction hearing device. Otol Neurotol.

[18] Rahne T, Plontke SK (2022). Systematic and audiological indication criteria for bone conduction devices and active middle ear implants. Hear Res.

[19] Rahne T, Seiwerth I, Gotze G (2015). Functional results after Bonebridge implantation in adults and children with conductive and mixed hearing loss. Eur Arch Otorhinolaryngol.

[20] Reinfeldt S, Hakansson B, Taghavi H (2014). Bone conduction hearing sensitivity in normal-hearing subjects: transcutaneous stimulation at BAHA vs BCI position. Int J Audiol.

[21] Schilde S, Plontke SK, Rahne T (2017). A three-dimensional geometric-morphometric study to quantify temporal bone growth and its consequences for the success of implanting bone anchored hearing devices. Otol Neurotol.

[22] Schraven SP, Gromann W, Rak K (2016). Long-term stability of the active middle-ear implant with floating-mass transducer technology: a single-center study. Otol Neurotol.

[23] Schwab B, Wimmer W, Severens JL (2020). Adverse events associated with bone-conduction and middle-ear implants: a systematic review. Eur Arch Otorhinolaryngol.

[24] Seiwerth I, Fröhlich L, Schilde S (2022). Clinical and functional results after implantation of the Bonebridge, a semi-implantable, active transcutaneous bone conduction device, in children and adults. Eur Arch Otorhinolaryngol.

[25] Seiwerth I, Schilde S, Wenzel C, Rahne T, Plontke SK (2021). Planungstools und Indikationen zur „virtuellen Chirurgie“ beim Knochenleitungssystem Bonebridge. HNO.

[26] Siegert R (2010). Combined reconstruction of congenital auricular atresia and severe microtia. Adv Otorhinolaryngol.

[27] Siegert R, Magritz R (2019). Otoplasty and auricular reconstruction. Facial Plast Surg.

[28] Spiegel JL, Kutsch L, Jakob M (2020). Long-term stability and functional outcome of an active middle ear implant regarding different coupling sites. Otol Neurotol.

[29] Sprinzl G, Lenarz T, Ernst A (2013). First European multicenter results with a new transcutaneous bone conduction hearing implant system: short-term safety and efficacy. Otol Neurotol.

[30] Sprinzl GM, Schoerg P, Muck S (2021). Long-term stability and safety of the soundbridge coupled to the round window. Laryngoscope.

[31] Sprinzl GM, Wolf-Magele A (2016). The Bonebridge bone conduction hearing implant: indication criteria, surgery and a systematic review of the literature. Clin Otolaryngol.

[32] Wang Y, Xing W, Liu T (2018). Simultaneous auricular reconstruction combined with bone bridge implantation-optimal surgical techniques in bilateral microtia with severe hearing impairment. Int J Pediatr Otorhinolaryngol.

[33] Weiss R, Leinung M, Baumann U (2017). Improvement of speech perception in quiet and in noise without decreasing localization abilities with the bone conduction device Bonebridge. Eur Arch Otorhinolaryngol.

[34] Wenzel C, Schilde S, Plontke SK (2020). Changes in bone conduction implant geometry improve the bone fit in mastoids of children and young adults. Otol Neurotol.

[35] Wickert E, Kurz A, Voelker J (2021). Simultane Implantation von Epithesenankern und Bonebridge zur Versorgung grosser Ohrmissbildungen. Laryngorhinootologie.

